# Fractal-induced 2D flexible net undulation

**DOI:** 10.1038/s41598-021-86418-5

**Published:** 2021-03-29

**Authors:** Michael Joon Seng Goh, Yeong Shiong Chiew, Ji Jinn Foo

**Affiliations:** Mechanical Engineering Department, School of Engineering, Monash University Malaysia, Subang Jaya, Malaysia

**Keywords:** Mechanical engineering, Engineering, Statistical physics, thermodynamics and nonlinear dynamics, Characterization and analytical techniques

## Abstract

A net immersed in fractal-induced turbulence exhibit a transient time-varying deformation. The anisotropic, inhomogeneous square fractal grid (SFG) generated flow interacts with the flexible net to manifest as visible cross-sectional undulations. We hypothesize that the net’s response may provide a surrogate in expressing local turbulent strength. This is analysed as root-mean-squared velocity fluctuations in the net, displaying intensity patterns dependent on the grid conformation and grid-net separation. The net’s fluctuation strength is found to increase closer to the turbulator with higher thickness ratio while presenting stronger fluctuations compared to regular-square-grid (RSG) of equivalent blockage-ratio, *σ*. Our findings demonstrate a novel application where 3D-reconstruction of submerged nets is used to experimentally contrast the turbulence generated by RSG and multilength scale SFGs across the channel cross-section. The net’s response shows the unique turbulence developed from SFGs can induce 9 × higher average excitation to a net when compared against RSG of similar *σ*.

## Introduction

Space-filling fractal grids are an interesting design made by repeating geometry at various scales. These grids have recently seen an increase in popularity for its use as turbulence generators^1–3^. Various studies have been conducted to investigate the effects of fractal grids as turbulators to improve thermal mixing^4,5^, in fluidic energy harvesting^6,7^, for burner combustion^8^, in impinging jets^9^, as well as other applications. To better understand this unique turbulence regime generated by fractal grids, numerical and experimental studies have been undertaken by many researchers. One of the unique properties attributed to fractal grid generated turbulence is the higher vorticity and turbulence intensities compared to regular grids^2^. A protracted turbulence generation region downstream of the grid followed by a distinct decaying region has been observed numerically^2,10,11^. For the channel centreline, this has also been documented experimentally^3,12,13^.

Typically, the experimental studies of fractal-induced turbulence have been concentrated on centreline measurements or require the use of costly particle imaging velocimetry (PIV) which are difficult to setup for full field measurements. As such, we endeavor to explore a possible new approach that allows for tomography-like characterization of the turbulent flow based on the direct fluid–structure interaction of the flow and a probing sheet. The concept of using a probing sheet is inspired by the response of particles or fibers in fluid flow that simultaneously expresses one or more characteristics of the flow^14–17^. Furthermore, the mathematical solutions for fiber and membrane dynamics in flow^18^ indicate a possibility of obtaining fluid flow perturbation from a fiber or membrane, in which case we decide to use a flexible net as the probing sheet. The first step on this journey will be the study of the probing sheet’s response to the flow dynamic which is recently made possible using videogrammetry^19^.

**Figure 1:**
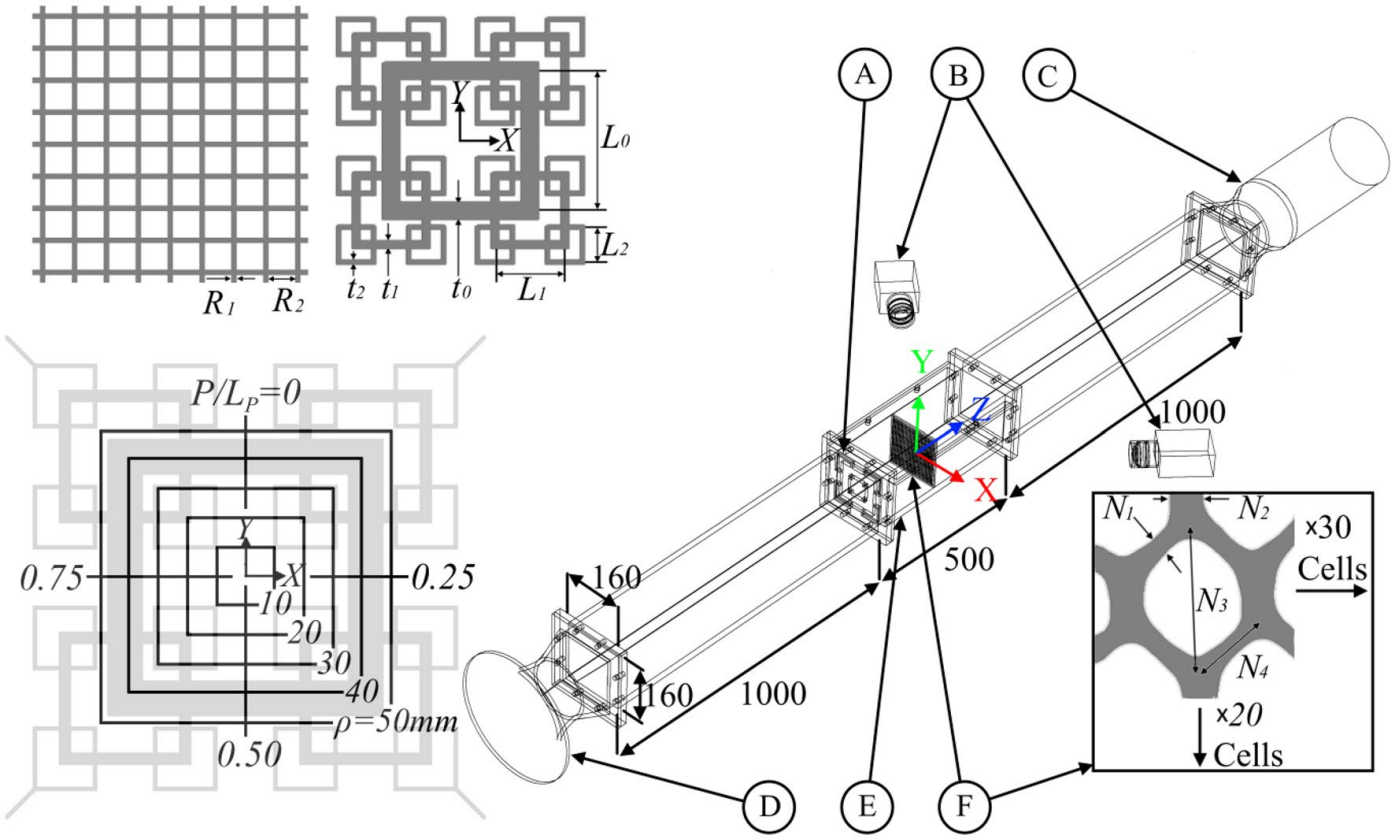
Clockwise from top left, regular and fractal square grid, experiment schematic, hexagonal cell net, concentric square coordinate system. Dimensions shown in Table 1. The experiment schematic consists of a grid/insert (**A**), two cameras (**B**), an axial draw-through fan (**C**), a bellmouth intake (**D**), the work area (**E**), and the hexagonal cell net (**F**). Note that the concentric square coordinate system introduces the parameter *ρ* which indicates the shortest distance from the square to its centre, and *P/L*_*P*_ a normalised parameter that varies from 0 to 1 along the square perimeter. (note: prepared in Paint.net 4.2.14, https://www.getpaint.net).

In the following, we demonstrate the use of a flexible net submerged in a flow domain for a comparative study of turbulence generated using a regular square grid (RSG) as control and square fractal grids (SFG) by analysis of the net’s physical response. A hexagonal celled polyester net secured in a thin rigid frame is placed at various distances streamwise from the tabulator. This allows us to have a better comprehension of the cross-sectional turbulence strength at various distances from the grid. Although we are still in the early stages of development and are some ways from directly detecting the flow’s turbulence strength, we can infer a comparative strength between the different turbulators based on the pattern and intensity of how the flow excites the net. Figure 1 shows the setup of our experiment with list of dimensions in Table 1.

**Table 1:** The dimensions for the grids and the net in Fig. 1.

Dimensions	SFG5	SFG6	SFG8
	mm		
*t*_*0*_	6.95	9.04	11.81
*t*_*1*_	3.11	3.54	4.05
*t*_*2*_	1.39	1.39	1.39
*L*_*0*_	80.0	80.0	80.0
*L*_*1*_	40.0	40.0	40.0
*L*_*2*_	20.0	20.0	20.0

Dimensions	mm		
*R*_*1*_	3.05		
*R*_*2*_	15.47		
*N*_*1*_	0.66		
*N*_*2*_	1.27		
*N*_*3*_	5.76		
*N*_*4*_	2.95		

## Methods

### Experimental setup and procedure

A 160 mm × 160 mm × 2500 mm transparent acrylic wind tunnel was setup with a bellmouth inlet and an axial drawthrough fan (Kruger, SG) at the outlet drawing air through the wind tunnel at a centreline flow velocity of *U*_*∞*_ = 5 ms^−1^ measured by a hotwire anemometer (Testo 405i, DE). As such, the flow Reynolds number, *Re*_*DH*_ is calculated to be 4.97 × 10^4^. The probing sheet is a hexagonal cell polyester net held in a rigid 1 mm Ø steel wire frame. The net is placed in the centre of the wind tunnel and imaged by two Pointgrey Grasshopper GS3-U3-15S5C-C industrial cameras at 45fps where each frame of the recording is buffered to memory before being stored for processing. The cameras’ internal and external parameters were determined by approximately 100 images of checkerboard calibration target with the Caltech Calibration Toolbox for Matlab^20^. The turbulator is placed upstream of the probing sheet with five turbulator-net distance which are 209 mm, 313 mm, 417 mm, 521 mm and 626 mm, hence which are equivalent to *L/t*_*2*_ = 150, 225, 300, 375 and 450 respectively. For each of the turbulator-net distance, the experiment is repeated five times to ensure sufficient data is captured for reliable and repeatable analysis.

### 3D time series reconstruction of net

The net was reconstructed using Neighbourhood Occupancy (NOCC) keypoints with Propagative Stereomatching^19^ for the first frame of the recording. Local DIC^21^ was then applied to obtain a point cloud of the net at each frame. Outlier elimination^22^ was then applied to each frame to ensure the net is a single smooth continuous surface before calculating the velocity, velocity fluctuation and root-mean-squared velocity fluctuation as well as subsequent computed quantities.

## Results and discussion

**Figure 2:**
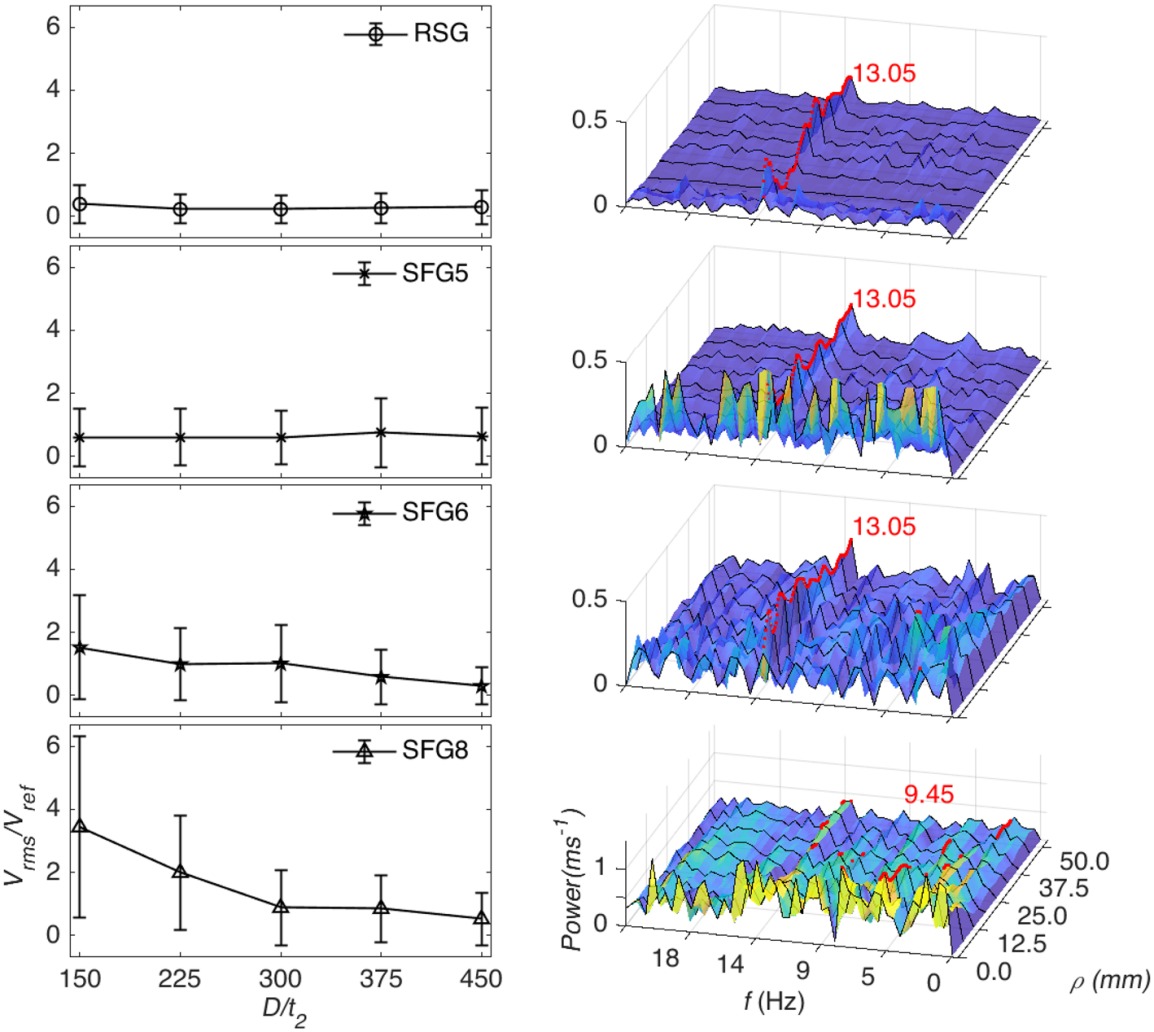
Left, mean and standard deviation of normalized *V*_*rms*_ against the streamwise distance, *L/t*_*2*_ of each insert. Right, power spectrum of each grid at all concentric square, *ρ* for turbulator distance, *L/t*_*2*_ = 150. Note: The peak at each *ρ* is shown as red dots with the most dominant frequency in red text. (note: prepared in Matlab R2018A, https://www.mathworks.com/products/matlab.html).

From the experiment, the time-series of the entire net’s fluctuation at each distance from the turbulator (i.e. grid-net distance or turbulator distance, *L*/*t*_*2*_) for each grid or insert design was successful captured. Due to the rich data gathered from the experiment, a more focused approach is employed to analyse the data by statistically summarising key quantities. Figure 2 presents the overall observation with the left side of the figure showing the root mean square (rms) velocity fluctuation of the net, *V*_*rms*_ normalised using the reference fluctuation velocity, *V*_*ref*_, which is calculated as the average *V*_*rms*_ of the net in a flow without turbulator. The immediately notable observation is that the average *V*_*rms*_/*V*_*ref*_ for RSG and SFG5 are roughly uniform with respect to distance from the turbulator. On the other hand, SFG6 and more prominently in SFG8, the average *V*_*rms*_/*V*_*ref*_ demonstrate a decay with *L*/*t*_*2*_. The standard deviations represented by the whiskers also indicate that the turbulence induced by the RSG interacts with the net to cause a rather uniform *V*_*rms*_/*V*_*ref*_ distribution across the surface. This uniformity is consistent at all distances from the turbulator. SFG5 displays a similar streamwise evolution albeit with a larger standard deviation, signifying a more varied amplitude response of the net independent of the turbulator distance with the exception of the downstream distance *L/t*_*2*_ = 375, as seen in Fig. 2. Applying a similar analysis, SFG6 and SFG8 both demonstrate that the standard deviation decays with turbulator distance. From the mean *V*_*rms*_/*V*_*ref*_, one can infer that the turbulence induced by SFG6 and SFG8 interact with the net more strongly when it is closer to the turbulator while the turbulence does not show significant decay for the RSG and SFG5. Such inference is further supported by the standard deviation where it is seen to reduce with *L*/*t*_*2*_ for SFG8, while SFG6 demonstrates a more gradual reduction of *V*_*rms*_/*V*_*ref*_ standard deviation with distance from the turbulator. The standard deviation for RSG and SFG5 are highly uniform with increasing *L*/*t*_*2*_ indicating the effect of the 2D cross-sectional grid-induced fluid flow undulation on the net does not vary greatly with distance.

From the results obtained, we are able to make a few inferences regarding the fluid–structure interaction between the grid-induced turbulence and the net. From the mean and standard deviation of the normalised *V*_*rms*_ evolution seen in Fig. 2, one can see that SFGs employed are able to generate a flow which is able to cause fluctuations in the net that are on average up to 9 times stronger across the surface despite having a smaller or similar blockage ratio as compared to the regular square grid employed. This is in line with numerical studies reported in literature^2,10,11^. The stronger net fluctuation indicates a stronger and more chaotic turbulence generated by the SFG which could be useful in numerous applications, e.g., in promoting an effective thermal mixing.

To make a more detailed comparison between the turbulators, in the right side of Fig. 2 we plot the frequency domain power spectrum of the net’s *V*_*rms*_ averaged along concentric squares at *L/t*_*2*_ = 150. Although at first glance, the power spectrum for the RSG seems unremarkable, we are able to observe a peak at ~ 13.05 Hz occurring at all concentric squares, *ρ*. Where *ρ* is the smallest distance of a concentric square centred from *x* = 0, *y* = 0, as defined in Fig. 1. The 13.05 Hz peak can also be seen in SFG5. However, as compared to RSG, we also see more energetic fluctuations in the lower frequencies at other *ρ*, especially around *ρ* < 10 mm. As we look into SFG6, it is seen that the overall chaotic dispersion of the power spectrum at all frequencies is comparable to the previously prominent 13.05 Hz peaks. Moreover, the high energy frequencies at *ρ* < 5 mm is not as obvious as with the SFG5, the peaks for a small number of *ρ* has also shifted to a frequency of 4.05 Hz (see Fig. 4 SFG5 and SFG6). In SFG8, and the peak at each *ρ* varies without a clear pattern ranging from 1.8 to 13.5 Hz across different *ρ* positions.

**Figure 3:**
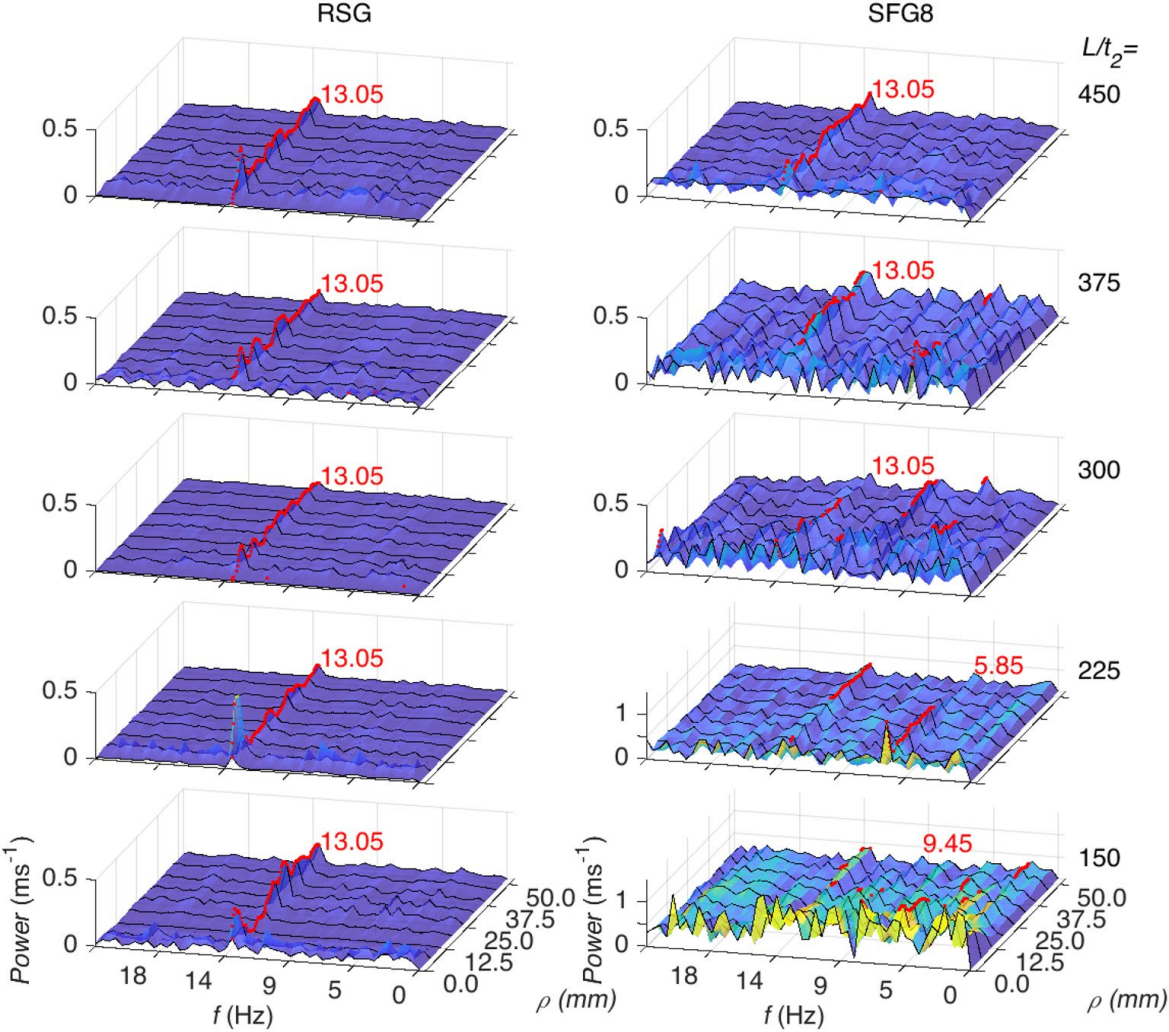
Power spectrum at each streamwise distance at all concentric square, *ρ* for RSG (left) and SFG8 (right). The peak of each *ρ* is shown as red dots with the most dominant indicated in red text. (note: prepared in Matlab R2018A, https://www.mathworks.com/products/matlab.html).

In Fig. 3, we take a look at the power spectrum plot at all turbulator distances for the weakest and strongest grid, namely, the control RSG and SFG8, respectively. For the former, we observe the same 13.05 Hz band marked in red at all turbulator distances. Furthermore, for all *L/t*_*2*_, there is a similar overall quantum of the power spectrum as reminiscence to the uniform standard deviation seen in Fig. 2. Looking into the distribution, the RSG at *L/t*_*2*_ = 150 the higher energy spots/peaks are well distributed across all frequencies and *ρ* with a slight bias towards the lower *ρ* around *ρ* < 5. As we move further to *L/t*_*2*_ = 225, a similar pattern where there is a slightly higher activity at concentric squares with a lower *ρ* is observed. At *L/t*_*2*_ = 300, the distribution is fairly homogeneous with no clear pattern to the power spectrum. At *L/t*_*2*_ = 375, we once again see the preference of lower *ρ* but a weaker and wider band going up to ~ *ρ* < 15 mm. Interestingly, it is observed that at *L/t*_*2*_ = 450 the more energetic frequencies once again reform at *ρ* < 10 mm.

Looking at the grid that induces the largest *V*_*rms*_ on the net, viz. SFG8, we see that at *L/t*_*2*_ = 150 and *L/t*_*2*_ = 225, the most dominant frequencies are 9.45 Hz and 5.85 Hz, respectively, although a strong 13.05 Hz fluctuations can be still be seen in the power spectrum. As seen in Fig. 2, at *L/t*_*2*_ = 150 the peak at each *ρ* varies widely, while for *L/t*_*2*_ = 225, the peaks concentrate along 5.85 Hz for *ρ* < 25 mm and 13.05 Hz for *ρ* > 25 mm. Interestingly, at *L/t*_*2*_ = 300 a wider distribution of the peaks compared to *L/t*_*2*_ = 225 can be observed ranging from 4.05 Hz to 13.5 Hz. Looking into *L/t*_*2*_ = 375, the majority of the peaks are at 13.05 Hz and 13.5 Hz with a number of peaks occurring at 4.05 Hz. Finally, at *L/t*_*2*_ = 450, 13.05 Hz mainly dominates the net fluctuation.

**Figure 4:**
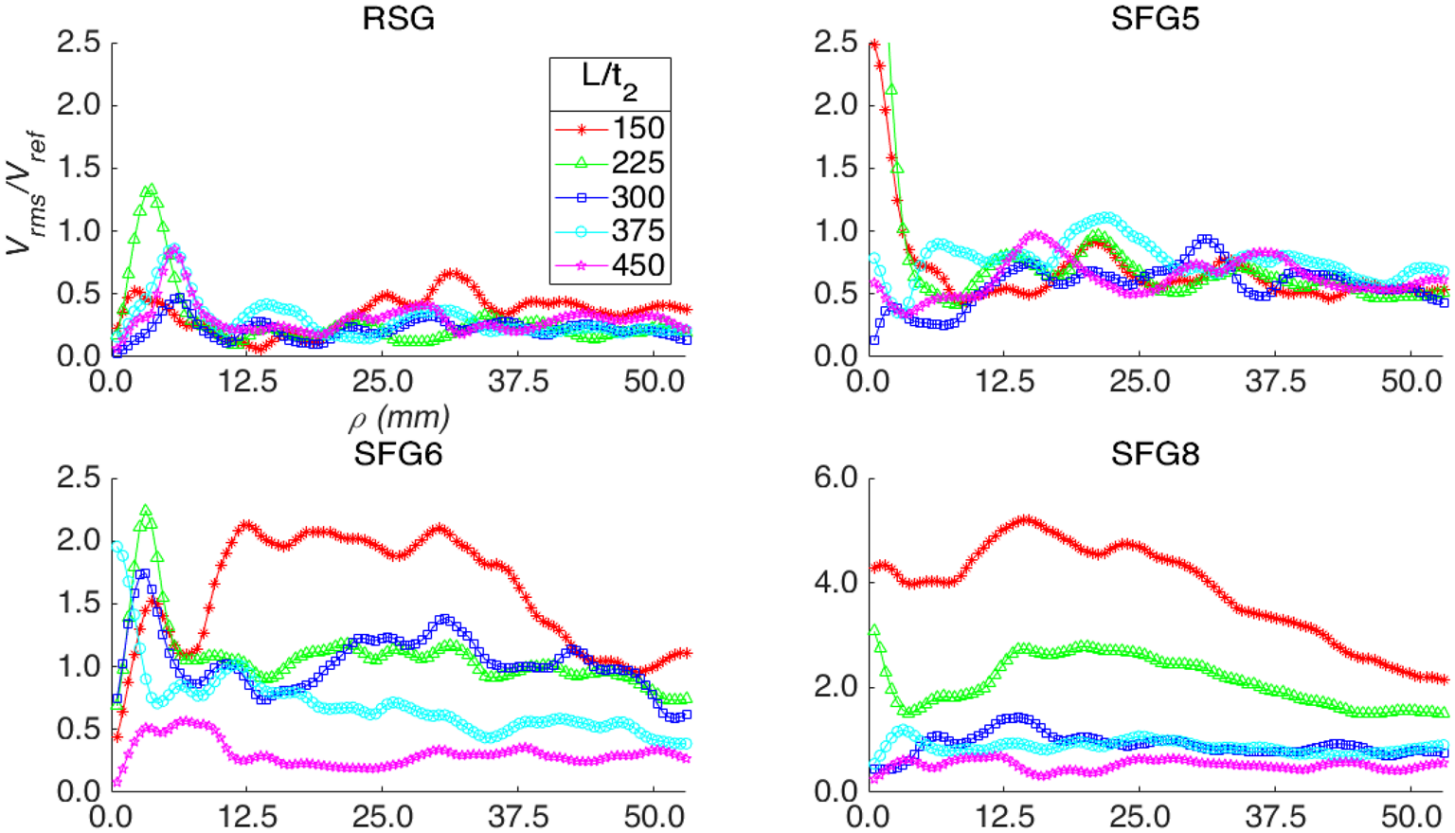
Normalized *V*_*rms*_ averaged across each concentric square, *ρ*. Note: The *Y*-axis scale for RSG, SFG5 and SFG6 is from 0 to 2.5 while SFG8 is 0 to 6. (note: prepared in Matlab R2018A, https://www.mathworks.com/products/matlab.html).

Based on the power spectrum analysis performed on the concentric square averaged velocity fluctuations in both Figs. 2 and 3, it is believed that the 13.05 Hz band across multiple *ρ* seen in most cases can possibly be attributed to resonance at the natural frequency of the system. This could be due to the periodic excitation of the constraint probing sheet resulting in resonance similar to flexible plate resonance in flow examined in literature ^23–25^. However, it should also be noted that the design and orientation of the probing sheet differs from the flexible plates in literature, which could also be a contributing factor to the difference in resonance frequency. Although more detailed investigation is required, this could potentially be used to tune various closed channel systems where resonance is desired. The variability of peak frequency with *ρ* in the power spectrum analysis for SFG8 in Fig. 3 could be an indication of inhomogeneity in the flow turbulence where vortices of various length-scales interact with the net differently. In which, we can clearly see that for SFG8, the turbulence pattern evolves with distance from the turbulator while it remains fairly unchanged for RSG. We hypothesise that for SFG8, the effect of the grid-induced turbulence on the net is significant enough to surpass the resonance of the system until *L/t*_*2*_ = 450 where the grid induced turbulence has decayed sufficiently that the net reverts to fluctuating at the system’s natural frequency. This is further supported by the fact that the mean and standard deviation of *V*_*rms*_/*V*_*ref*_ of SFG8 and RSG at *L/t*_*2*_ = 450 are fairly similar. In Fig. 4, the strongest fluctuations are mostly observed to be at *ρ* < 10 mm for RSG, SFG5 and SFG6, in many cases being significantly large than at the largest concentric squares. This may be due to the design of the probing sheet where it is held rigidly at the edges while the strands towards the centre of the net are more unrestraint and allowed more freedom of motion.

From the observation made where a high power band of frequencies is observed between certain concentric squares in various cases, we plot the average *V*_*rms*_/*V*_*ref*_ along each *ρ* for all grids and *L/t*_*2*_ in Fig. 4 to investigate the local net’s fluctuation. For the control RSG, It is seen that the overall net perturbation is quite flat with average *V*_*rms*_/*V*_*ref*_ for the squares at each *ρ* staying mostly between 0.05 and 0.4. Interestingly, peaks are observed around *ρ* = 7 mm for all turbulator distances. This indicates that net is experiencing a strong fluctuation in the central region of the net while the rest of the net undergoes a rather uniform low undulation. In SFG5, very strong peaks are harvested near the centre of the net similar to that of RSG. However, as compared to RSG, SFG5 demonstrates a stronger overall fluctuation with noticeable concentration around *ρ* = 13 to *ρ* = 22. For SFG6, we once again secure stronger fluctuations near the centre portion of the net. Nevertheless, at *L/t*_*2*_ = 150 strong fluctuations can be seen from about 12 to 36 mm significantly higher than at other turbulator distance. It can also be seen that for SFG6, there is a clear distinction between the fluctuation strength of the net for *ρ* > 8 mm at most turbulator distance, of which, it is clearly seen that the fluctuation is stronger the closer the net is placed to the turbulator with the exception of *L/t*_*2*_ = 225 and *L/t*_*2*_ = 300 which do not show clear separation. Lastly, as we look into SFG8, the distinction between the fluctuation strength is clear for most *ρ*. However, in SFG8, the difference in fluctuation strength at *L/t*_*2*_ = 300 and *L/t*_*2*_ = 375 is not immediately obvious. Another unique observation for SFG8 is that the previously observed preference towards strong fluctuations at the centre of the net appears to weaken especially for the smallest turbulator distance *L/t*_*2*_ = 150, in comparison with SFG5.

**Figure 5:**
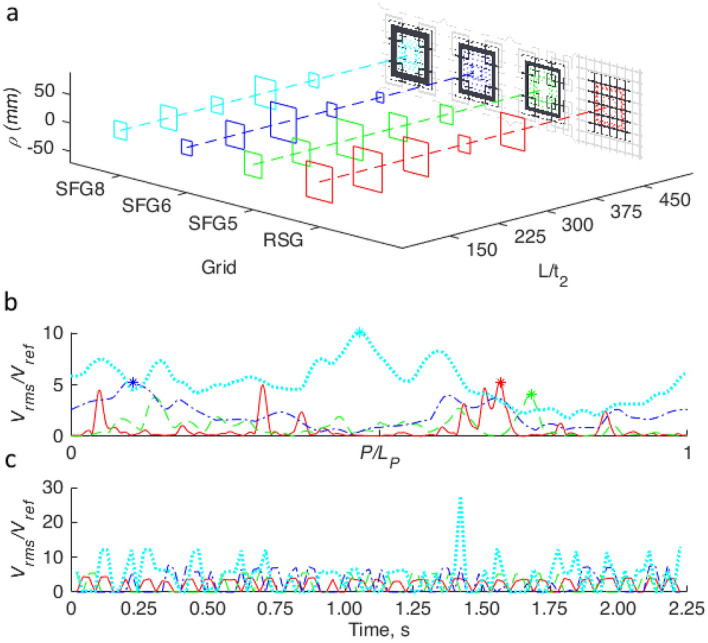
(**a**) Concentric square, *ρ* with the largest *ρ* averaged *V*_*rms*_ for each grid at each streamwise distance *L/t*_*2*_. (**b**) Normalized *V*_*rms*_ profile of maximum concentric square at *L/t*_*2*_ = 150, with maximum point indicated by asterisk. (**c**) Normalized *V*_*rms*_ time-series of the corresponding maximal in (**b**). (note: prepared in Matlab R2018A, https://www.mathworks.com/products/matlab.html).

To further analyse the evolution of the net’s response along different turbulator distances, we visualise in Fig. 5 the strongest squares that are at *ρ* > 8 mm. For RSG, we can see that the strongest square is quite consistent for all *L/t*_*2*_ being around *ρ≈*30 mm. Although, at *L/t*_*2*_ = 375 the strongest square is at *ρ* = 13 mm. We notice an interesting pattern in SFG5 where the strongest square starts small around *ρ* = 20 mm closest to the turbulator, gradually increases to *ρ* = 31 mm at *L/t*_*2*_ = 300 before decreasing again to *ρ* = 15 mm, furthest from the turbulator. A similar pattern is observed with SFG6. However, in SFG6 the increase and decrease is more substantial; going from *ρ* = 13 mm at *L/t*_*2*_ = 150 to *ρ* = 31 mm at *L/t*_*2*_ = 300 and down to *ρ* = 8 mm at *L/t*_*2*_ = 450. SFG8 presents an alternating pattern where the strongest squares are *ρ* = 15, 20, 14, 25 and 12 mm at *L/t*_*2*_ = 150, 225, 300, 375 and 450, respectively.

We then plot the time averaged normalised *V*_*rms*_ profile of the strongest square at *L/t*_*2*_ = 150 for each grid in Fig. 5b. Here *P* denotes the position along the square starting from *x* = 0, *y* = *ρ* and *L*_*P*_ is the perimeter length of the square. It can be observed that the profile for RSG is quite flat close to *V*_*rms*_*/V*_*ref*_ = 0, with sporadic peaks at various points along the square. SFG5 demonstrates a similar profile where it is largely flat with sporadic peaks although the peaks tend to be wider and occur less frequently as compared to RSG. SFG6 on the other hand has less pronounced peaks where *V*_*rms*_*/V*_*ref*_ varies gently from 0 to 5. Finally, SFG8 induces the most fluctuation with a minimum of *V*_*rms*_*/V*_*ref*_ = 1.8 and a maximum of *V*_*rms*_*/V*_*ref*_ = 10.1. Similar to SFG5 and SFG6, the width of the peaks in SFG8 are clearly wider in comparing with the control RSG. At the strongest point along the profile, we further analyse the effect of the turbulator on the net by looking at the time series of the net’s fluctuation as *V*_*rms*_*/V*_*ref*_. Figure 5c shows the time-series of the strongest point on the net of each grid at *L/t*_*2*_ = 150. From Fig. 5c an immediate observation that we can make is that RSG has a highly regular *V*_*rms*_*/V*_*ref*_ fluctuation pattern with consistent frequency and amplitude. SFG5 also demonstrates consistent amplitude about *V*_*rms*_*/V*_*ref*_ = 2 higher than RSG but the frequency is less consistent compared to RSG as we can see some regions where little or no fluctuation is observed. In SFG6 the amplitude is still largely consistent about *V*_*rms*_*/V*_*ref*_ = 2 higher than SFG5. Similar to SFG5, SFG6 has some regions of low fluctuations although no clear pattern can be seen as to when the low fluctuations occur. Finally in SFG8 the time-series is a lot more chaotic with a mix of amplitude, frequency and peak width. This indicates increasing disorder as we go from RSG, SFG5, SFG6 to SFG8.

**Figure 6:**
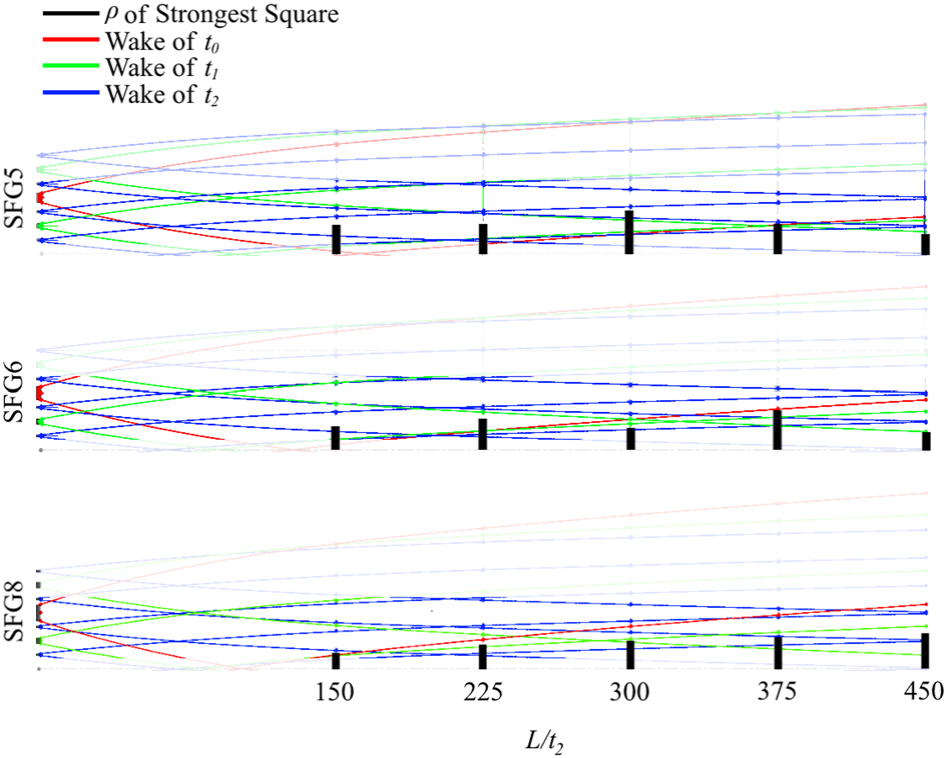
Wake of fractal grid bars superimposed for each bar thickness. (note: prepared in Paint.net 4.2.14, https://www.getpaint.net).

Although further investigation is needed to ascertain the mechanism behind the observation made in Fig. 5, wake interactions could provide a possible explanation. We hypothesise that multiple regions of high fluctuations may be present for each cross-section. This is supported by the observation of various *ρ* with high fluctuations in Fig. 4 as well as numerical studies reported in literature showing more than one region of strong fluctuation at each streamwise evolution^2,26^. This could be due to the complex interaction of wakes from two or more bars in a manner similar to the centreline wake interaction proposed in literature^3^. Figure 6 illustrates this concept where the top half of a SFG slice is shown with the typical wake width, *w* at a streamwise distance, *L* estimated as $w \sim \sqrt {t_{j} L}$ ^3^ based on the thickness of the bar, *t*. The wake from each of the bars can be seen to encounter wakes from other bars multiple times at various streamwise distances, in some cases such as around *L/t*_*2*_ = 166 of SFG8, it can even be seen that the wake from three different thickness bars (*t*_*0*_, *t*_*1*_ and *t*_*2*_) could interact. In many cases, the *ρ* with the strongest fluctuation is near to positions where multiple lines converge. However, it is important to note that this illustration serves only to conceptualise the potential mechanism behind the present observation. In actuality, the interactions are far more complex when looking beyond just a slice of the SFG. An example of this can be seen in literature where the orientation and geometry of the fractal strongly affects the turbulence wake^27^. Moreover, the wake width estimation calculation used above does not account for interaction with other wakes upstream of *L*. Although numerous numerical and experimental studies with fractal-generated turbulence has been performed, it is believed there is opportunity for future research into furthering the understanding of the complex interaction of multilength-scale wakes generated by different bars of different thickness, orientation and connection (e.g. corners of each SFG iteration or cross between different iterations).

**Figure 7:**
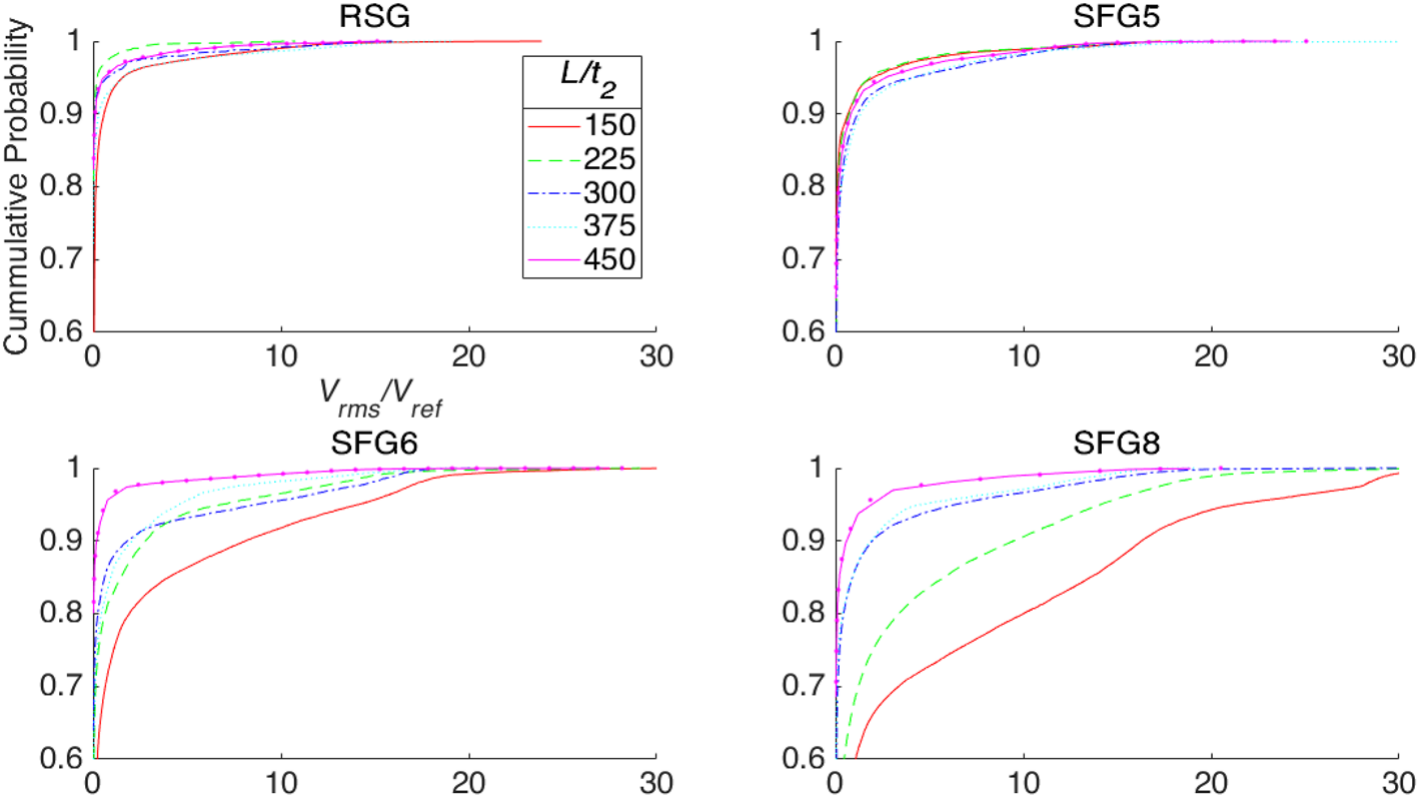
Cummulative probability of each 2D planar space-filling insert’s normalized *V*_*rms*_. (note: prepared in Matlab R2018A, https://www.mathworks.com/products/matlab.html).

The cumulative probability distribution of the normalised velocity fluctuation at the strongest point is plotted to compare the turbulators at *L/t*_*2*_. As shown in Fig. 7, it is immediately apparent that RSG has a very flat profile where about 95% of the net has a low velocity fluctuation of *V*_*rms*_*/V*_*ref*_ < 0.9 while ~ 99% of the fluctuations are below *V*_*rms*_*/V*_*ref*_ = 6. The cumulative probability distribution is also highly similar for all distances except at *L/t*_*2*_ = 150, which shows more fluctuation between *V*_*rms*_*/V*_*ref*_ = 5 to 10. Looking at SFG5, the cumulative probability distribution varies slightly with distance from the turbulator. Yet, as compared to the RSG the net has a stronger fluctuation where ~ 95% of the fluctuations have a value of *V*_*rms*_*/V*_*ref*_ < 2.7. Interestingly, approximately 99% of the fluctuations do not go over *V*_*rms*_*/V*_*ref*_ = 11. Moving on to SFG6 and SFG8, there is clear distinction between the cumulative distribution plots at different distance from the turbulator. We observe that the closer the net is to the turbulator, the more likely it is to observe large fluctuations. For instance, at *L/t*_*2*_ = 150, 95% of the fluctuations are below *V*_*rms*_*/V*_*ref*_ = 14 while the top 1 percentile has *V*_*rms*_*/V*_*ref*_ > 19, on the other hand, at the next position of *L/t*_*2*_ = 225, 95% of the fluctuations are below *V*_*rms*_*/V*_*ref*_ = 6, while 99% are not more that *V*_*rms*_*/V*_*ref*_ = 16. This is even more pronounce in SFG8, where the top 5 percentile has fluctuations of *V*_*rms*_*/V*_*ref*_ > 21, while the top percentile fluctuates above *V*_*rms*_*/V*_*re*_ = 29 at *L/t*_*2*_ = 150. At *L/t*_*2*_ = 225, this reduces to *V*_*rms*_*/V*_*ref*_ > 14 and *V*_*rms*_*/V*_*ref*_ > 20 at the top 5 and 1 percentile, respectively.

In short, SFG-generated turbulence presents unique advantages over RSGs as investigated by the response of a net when subjected to the grid-induced turbulence. As neatly summarised in Fig. 6, we can see that SFG8 which is of comparable blockage ratio to the RSG is able to cause the net to respond with a significantly wider range of fluctuations. The cumulative probability of normalised *V*_*rms*_ in Fig. 7 further highlights the uniqueness of SFGs, where both of the SFG8 and SFG6 demonstrate turbulator distance dependency of the net’s range of fluctuations. This is in stark contrast to RSG where the cumulative probability plots are highly similar at all 5 turbulator distances investigated. We believe this could be due to the unique turbulence regime of SFG where the net’s response varies with its location in the generation region and decay region described in literature^1,2^.

## Conclusion

The transient time-varying deformation exhibited by a net immersed in fractal-induced turbulence was proposed as a means to provide insights into the turbulent flow’s characteristics. This was demonstrated by comparing the resultant flow from an RSG against the flow resulting from various SFGs. The net’s fluctuation at each turbulator-net distance was able to reveal the decay region of SFGs as described in experimental and numerical literature^1–3,10,11^. Furthermore, the net also supports the finding by other researchers where the turbulence increases with increase in SFG bar thickness^1–3^ where in our experiment, SFG8 induces the strongest turbulence followed by SFG6 and SFG5 has the weakest turbulence. SFG8 was also found to generate turbulence up to nine times stronger compared to RSG which has a comparable blockage ratio. The usage of a net as probing sheet also allowed for new insights to be gained where wake interactions may have led to the observed growing and shrinking concentric squares of strongest fluctuation.

Unequivocally, the employment of a net to qualitatively and quantitatively compare grid turbulators could potentially help bring fractal grids into more mainstream applications by allowing quick in-situ assessment of the turbulence generated by the grids.
